# Using standard treatment and offloading principles to heal a wound of a patient who ambulates upon “all fours”

**DOI:** 10.1186/1757-1146-5-S1-P3

**Published:** 2012-04-10

**Authors:** Lindy Begg, Patrick McLaughlin, Karin Sutton, Thomas Daly, Mauro Vicaretti, Joshua Burns

**Affiliations:** 1 Foot Wound Clinic, Department of Surgery, Westmead Hospital, NSW, 2145, Australia; 2 School of Biomedical and Health Sciences, Faculty of Health, Engineering and Science, Victoria University, Melbourne 8001, Australia; 3 Institute of Sport, Exercise and Active Living, Victoria University, Melbourne, 8001, Australia; 4 The Podiatry Clinic, 51 Station Street, Wentworthville, NSW, 2145, Australia; 5 Institute for Neuroscience and Muscle Research, The Children’s Hospital at Westmead/Paediatric Gait Analysis Service of New South Wales/Faculty of Health Sciences, The University of Sydney, NSW, 2145, Australia

## Background

A 71 year old, weighing 80 kg was referred to the Foot Wound Clinic despite not having feet. The patient had suffered a traumatic Above Knee Amputation of the right limb and an Above Knee Amputation of the left limb from the same incident in 1969. The patient ambulates on “all fours” or upon the femurs alone and continues to work full-time as a landscaper. The patient presented for review of a wound over the right stump with the expectation that he would undergo surgical debridement and a skin graft. The patient had adequate arterial flow therefore with standard wound care and offloading, healing should ensure. The patient has been referred to Rehabilitation Services for review and in the interim consented to being treated with a Total Contact Cast (TCC).

## Materials and methods

Pressure to the stumps was assessed using emed^®^ (novel Gmbh, Germany). A total contact cast incorporating 6 mm slow-rebound cellular urethane and 6 mm soft cellular urethane inlay as described previously [1] was fabricated for the right stump. The TCC was removed and a capacitance sensor insole (pedar^®^, novel Gmbh, Germany) was placed within the cast measuring medially to laterally including the wound site.

## Results

The area of maximum pressure was 54 cm^2^ and peak pressure was 425 kPa at the stump of the right femur using the emed^®^; average maximum pressure indicated that pressure was born at the medial to lateral area of the stump with less than 15 kPa recorded at the wound site using the pedar^®^. The VAS score of 7 was reported prior to the TCC and 0 following the intervention. The patient reported an increase in activity levels.

## Conclusions

Healing is imminent due to the femur being held in suspension within the TCC. This case history highlights that a challenging patient notwithstanding; standard assessment and intervention is essential.
